# Pathways from Cannabis to Psychosis: A Review of the Evidence

**DOI:** 10.3389/fpsyt.2013.00128

**Published:** 2013-10-14

**Authors:** Jonathan K. Burns

**Affiliations:** ^1^Department of Psychiatry, Nelson R Mandela School of Medicine, University of KwaZulu-Natal, Durban, South Africa

**Keywords:** *Cannabis*, psychosis, schizophrenia, causality, neurobiology, cognition, outcome

## Abstract

The nature of the relationship between cannabis use (CU) and psychosis is complex and remains unclear. Researchers and clinicians remain divided regarding key issues such as whether or not cannabis is an independent cause of psychosis and schizophrenia. This paper reviews the field in detail, examining questions of causality, the neurobiological basis for such causality and for differential inter-individual risk, the clinical and cognitive features of psychosis in cannabis users, and patterns of course and outcome of psychosis in the context of CU. The author proposes two major pathways from cannabis to psychosis based on a differentiation between early-initiated lifelong CU and a scenario where vulnerable individuals without a lifelong pattern of use consume cannabis over a relatively brief period of time just prior to psychosis onset. Additional key factors determining the clinical and neurobiological manifestation of psychosis as well as course and outcome in cannabis users include: underlying genetic and developmental vulnerability to schizophrenia-spectrum disorders; and whether or not CU ceases or continues after the onset of psychosis. Finally, methodological guidelines are presented for future research aimed at both elucidating the pathways that lead from cannabis to psychosis and clarifying the long-term outcome of the disorder in those who have a history of using cannabis.

## Introduction and Historical Perspective

*Cannabis sativa* is the most widely used drug in the world and archeological evidence from China indicates that humans used cannabis as early as 4000 BCE (1). An association between CU and mental illness, in particular psychotic illness, was recognized as early as 1895 in a report by the Indian Hemp Drugs Commission (2).

It is worth noting several extracts from this report, as they suggest that clinicians working more than a century ago, were similarly uncertain regarding the nature of the observed association between cannabis and psychosis. Curiously, it appears from these extracts that our clinical forbearers were indeed conscious of several fundamental issues which modern science is only now confirming in relation to the effects of cannabis on the human brain. In the twelfth chapter, the report (2) states:

In relation to a causal relationship…

In answering the question, therefore, on what the evidence rests that hemp drugs may induce mental aberration, the Commission would offer the following remarks: The evidence may be considered under two heads – (a) popular; (b) scientific. The popular idea that the use of hemp drugs may induce insanity can be traced back for many centuries, and the present day views on the subject are no doubt the outcome of old popular ideas which have been handed down and become concrete.

But… we have a number of instances where the hemp drug habit has been so established in relation to the insanity that, admitting (as we must admit) that hemp drugs as intoxicants cause more or less of cerebral stimulation, it may be accepted as reasonably proved, in the absence of evidence of other cause, that hemp drugs do cause insanity.

In relation to underlying biological processes…

The acute symptoms correspond to the temporary saturation of the body with the poison, while the chronic symptoms are the expression of definite anatomical lesions in the brain gradually developed under toxic influence… Further, in regard to what has been said about hemp drug mania, it may be noted that it is not improbable, though it has not been established by evidence, that prolonged abuse of the drugs may give rise in some cases to definite brain lesions resulting in a progressive weakening of all the faculties leading to dementia.

In relation to differential vulnerability to psychosis…

In respect to the alleged mental effects of the drugs. It may indeed be accepted that in the case of specially marked neurotic diathesis, even the moderate use may produce mental injury… The individual factor with its idiosyncrasies plays here, as everywhere, a very important part… Nervous and predisposed persons appear to be more easily affected than normal subjects.

In relation to specificity of symptoms of psychosis in cannabis users…

The evidence obtained by the Commission appears to indicate that in the cases of alleged hemp drug insanity which find their way into asylums, there are no typical features in the premonitory symptoms and no pathognomonic symptoms in the insane condition on which to base a determination of causation… The majority of medical witnesses who have studied the subject are clearly of opinion that there is nothing typical in the symptomatology of hemp drug mania to distinguish it from mania due to other causes. But at the same time several express an opinion that the symptoms are of shorter duration in hemp drug mania than in mania due to other causes… The careful inquiry which has been made by the Commission into all the alleged hemp drugs cases admitted in one year into asylums in British India demonstrates conclusively that the usual mode of differentiating between hemp drug insanity and ordinary mania was in the highest degree uncertain, and therefore fallacious.

Interestingly, it seems that clinicians in British India encountered the same difficulties in establishing cannabis as the causal agent in cases of cannabis-using individuals presenting with psychotic illness. In fact, as occurs too often in contemporary clinical practice, this difficulty also led clinicians in that era to resort to over-diagnosis of cannabis-induced psychosis, as is evident in the following extract:

Surgeon-Major Willcocks, of Agra, says: “Ordinarily it has been the practice to enter hemp drugs as the cause of insanity where it has been shown that the patient used these drugs. I cannot say precisely why this is the practice. It has come down as the traditional practice.”

Reference to this historical enquiry into the relationship between cannabis and psychosis, highlights a number of key issues that lie at the center of modern research in this field. In reviewing the relationship between cannabis and psychosis, there are six questions that need to be addressed:

1. Is cannabis an independent cause of psychosis?
2. If so, what are the neurobiological processes underlying this causal relationship?
3. Is the risk for psychosis the same in all individuals using cannabis; and if not, is there a neurobiological explanation for differential risk?
4. Are there specific clinical features of psychosis in cannabis users that differentiates them from psychosis in non-cannabis users?
5. What are the cognitive effects of cannabis use in individuals with psychosis?
6. Does cannabis use impact on course and outcome in individuals with psychosis?

In this review, each of these questions will be addressed and the relevant accumulated evidence presented. In conclusion, I will consider the issue of whether there might be different pathways from cannabis use (CU) to psychotic illness; and present an evidence-based hypothesis that will hopefully offer some direction for future research in this field.

## Cannabis and Risk for Psychosis – A Causal Relationship?

In examining the evidence for a causal relationship between cannabis and psychosis, it is important to acknowledge a number of limitations that are inherent in this research field. The first is the matter of definitions. Published studies vary in terms of the population upon which they focus – some studies limit their inclusion criteria to a narrow definition of schizophrenia, while others include a broad definition of psychotic disorders. Similarly, there is marked variability between studies in terms of defining “CU” (3). CU may be defined as current use or recent use or lifetime use; and within these categories there are further differences in definition. For example, recent use may be regarded as: use within the last month; daily use for at least the last month; weekly use for at least the last month; or as varying frequencies of use over the last 3 months or even 6 months. Other limitations include: most studies measure CU based on self-report, which tends to be associated with under-reporting; self-report of CU is subject to recall bias; in most cases, there is limited information on other substance use, so that analyses are often unable to control for the confounding effects of other substances such as stimulants; it is often difficult to control for factors such as potency of cannabis, frequency of use, and amount of cannabis consumed, due to statistical power issues; and finally and perhaps most importantly, most studies are unable to demonstrate temporal priority of cannabis in relation to early prodromal features of psychosis [the Dunedin study is one of the few that have achieved this methodologically (4, 5)].

These limitations are highly relevant in attempting to establish a causal relationship between “CU” and “psychosis.” This is because any attempt to establish “causation” must fulfill the following criteria as defined by Susser (6): association; temporal priority; and direction (where the last implies that changes in the putative cause will actually lead to changes in the outcome, and that the association between putative cause and outcome does not derive from a third factor common to both) (5). Other criteria for causation listed by Hill (7) include: strength (i.e., a dose-response relationship); consistency; specificity; biological gradient; temporality; coherence; and plausibility (i.e., a plausible biological mechanism linking exposure and outcome). Thus the evidence-base on cannabis and psychosis should at least satisfy the majority of these criteria, and must meet the criterion of temporality which, according to Rothman and Greenland (8), is the *sine qua non-for* causality (5).

Multiple studies confirm that CU is approximately two times more frequent among people with schizophrenia than in the general population (9, 10). Furthermore, CU is considered a significant risk factor for both suicide attempts and behavior in psychotic samples (11). This raises the question of whether cannabis plays an etiological role in the onset of schizophrenia, or whether people with schizophrenia are prone to increased use of cannabis. Studies of retrospective reports on CU typically show that approximately one third of individuals commence CU prior to onset of psychotic illness (12, 13). Retrospective studies are subject to recall bias; thus prospective data is required to confirm temporal priority (and thus causality) of CU. A number of systematic reviews have focused on prospective studies only with longitudinal designs and these report pooled odds ratios varying between 1.41 and 2.34 (5, 14, 15). Henquet et al. (14), whose analysis arrived at a pooled odds ratio of 2.1, noted that this result held regardless of whether studies with narrow clinical outcome were included (OR: 2.4) or whether those with broader outcomes were included (OR: 1.9). Interestingly, Arseneault et al. (5) who arrived at a pooled odds ratio of 2.34, included a very narrow definition of clinical outcomes; while Moore et al. (15), who arrived at a pooled odds ratio of 1.41 in their systematic review, included a very broad definition of psychotic outcomes. The impression therefore from these systematic reviews is that narrow definitions of psychosis (i.e., limited to non-affective psychosis/schizophrenia-spectrum) are associated with slightly higher odds ratios of approximately 2.3–2.4; while broader definitions are associated with slightly lower odds ratios of approximately 1.4–1.9. Notably, all studies included in these three major systematic reviews adjusted for a range of confounding factors. In summary, these reviews suggest that CU is associated with roughly a twofold increased risk of developing psychosis (specifically non-affective, schizophrenia-spectrum disorders), thereby confirming an association between exposure and outcome.

Temporal priority of CU was confirmed in at least two studies which showed that CU during early adolescence increases the risk for later non-affective psychosis outcome (4, 16). In the Dunedin Study in New Zealand, a general population birth cohort of 1037 individuals were assessed at age 11 for psychotic symptoms, at ages 15 and 18 for self-reported CU, and at age 26 for schizophrenia and schizophreniform disorder outcomes (4, 5). Thus, controlling for psychotic symptoms at baseline, the authors were able to show an association between CU at ages 15 and 18 and increased risk for psychotic symptoms at age 26 years. Early CU (by age 15) was associated with a threefold increased risk of schizophreniform disorder at age 26 years (thus confirming temporal priority); but was not associated with later depressive outcomes (thereby indicating specificity of outcome). The use of other drugs in adolescence did not predict psychotic outcomes over and above the effect of CU (indicating specificity of the exposure) (5). The Dutch NEMESIS study (10) as well as the Swedish conscript follow-up study (17) both demonstrated a dose-response relationship between increased CU and increased risk of later psychosis – in the NEMESIS study, the highest risk (OR: 6.8) was associated with the highest level of CU.

In conclusion, there is good scientific evidence, emanating from a number of key studies involving careful longitudinal designs, to conclude that a causal relationship does exist between CU and psychotic illness. Specifically, these studies suggest that this relationship exists in relation to non-affective schizophrenia-spectrum disorders. These studies have demonstrated most of the key criteria for establishing causality, namely: association; temporal priority; specificity; and strength (dose-response relationship). What remains to be considered is the question of plausibility – is there a plausible biological mechanism that could explain the etiological role of cannabis in psychosis and schizophrenia?

## The Neurobiological Basis of Cannabis as a Cause of Psychosis

In the early 1990s, the cannabinoid (CB) receptors were genetically determined – the distribution of CB_1_ was mapped in high densities to the striatum, hippocampus, and cerebellum; and in moderate to low densities to the amygdala, midbrain, and cerebral cortex (18). CB_1_ are situated on presynaptic terminals that release GABA, glutamate, serotonin, dopamine, and Ach; and interaction between this receptor and its endogenous endocannabinoid ligands (e.g., anandamide) results in limiting of neurotransmitter release (19). Thus this system plays an important role in maintaining and determining synaptic plasticity. Importantly, endocannabinoid signaling is present during gestation and early infancy and plays a critical role in neuronal proliferation, migration, axonal guidance, positioning of cortical interneurons, and synaptogenesis (20). Experimental aberrations in endocannabinoid signaling during critical periods result in significant disruptions in neurodevelopment. While the role of endocannabinoid signaling during adolescence has not been fully elucidated, one may reasonably assume that the neurodevelopmental role of this system continues during adolescence when regions such as the hippocampus and prefrontal cortex are still undergoing marked development (18). Notably too, CB_1_ expression patterns increase dramatically throughout the adolescent period in areas including the frontal cortex, striatum, and hippocampus (21). Likewise, there appear to be peaks in endocannabinoid levels during the adolescent period (22, 23). Thus, as Malone et al. (18) conclude, “endocannabinoid signaling is an important determinant of maturation of the adult brain… it seems quite likely that disruption of normative endocannabinoid signaling during adolescence may have long-standing consequences on adult brain function.” Animal models show that early exposure to CB agonists result in a variety of mostly cognitive deficits in adult animals, including working memory dysfunction, disruption in pre-pulse inhibition of startle (a measure of sensory gating), a significant decrease in social behavior and increased locomotor activity (24–26). In summary, these observations have led to the so-called “endocannabinoid hypothesis of schizophrenia” (27).

Disruption of the CB system during development impacts on several other neurotransmitter systems, notably the GABA and dopamine systems. GABAergic neurons in the prefrontal cortex are rich in CB_1_ receptors which, when activated, result in a decrease in extracellular GABA release (28). It has been hypothesized that repeated exposure to cannabis during adolescence may alter the balance of GABAergic inhibitory inputs to pyramidal neurons in the prefrontal cortex that could lead to impaired cognitive function (29). Furthermore, CU leads to increased extracellular dopamine; probably through the activation of CB_1_ receptors on GABAergic interneurons, which in turn disinhibit dopaminergic neurons (30). Kapur (31) has argued that psychosis results from aberrant reward prediction and abnormal attribution of salience caused by disordered dopamine transmission; while Laruelle (32) postulated that the dopaminergic abnormalities associated with schizophrenia are due to “dopamine sensitization” beginning in adolescence. Dopamine sensitization has been suggested in relation to the links between early stress and trauma (e.g., childhood sexual abuse) and the observed increased risk for schizophrenia (33). Evidence supporting the role of dopamine sensitization in CU, comes from a study by Houston et al. (34) where a significant interaction was found between early exposure to cannabis and childhood sexual trauma on psychosis outcome. Importantly, in this study, no main effect was observed for either sexual trauma or CU on psychosis outcome; suggesting that previous exposure to stress sensitizes individuals, so that subsequent life stresses evoke progressively greater responses over time (i.e., in this case there is cross-sensitization between early stress and cannabis) (35). In terms of dopamine sensitization, the individual eventually reaches a lasting state of dopamine dysregulation (36, 37). Henquet et al. (35) note that the dose-response relationship between CU and risk for psychosis suggests a dopamine sensitization process.

## The Biological Basis of Differential Risk for Psychosis in Cannabis Users

Cannabis is the most widely used drug in the world – millions of people use it – however, only a small proportion of users develop psychotic illness. This suggests that individual genetic factors must play a role in altering individual susceptibility to the psychotic-inducing potential of cannabis; thus gene-environment interactions are implied.

There are two ways to measure genetic liability to psychosis – directly and indirectly (14). Indirect measurement involves the use of individuals who are shown to exhibit liability to psychosis (measured using psychosis proneness scales) or are liable to psychosis by virtue of being first degree relatives of psychotic probands. Individuals measured as liable to psychosis are at greater risk of developing cannabis-induced psychotic experiences during the flow of everyday life (38); and their 3–5 year risk of developing psychotic symptoms while using cannabis is 51% compared with 21% in those using cannabis who do not show psychosis liability (39). A family study by McGuire et al. (40) found that patients who developed acute psychosis after using cannabis were more likely to have a positive family history of schizophrenia than those patients who screened negative for CU.

Unlike indirect measures of genetic risk which rely on psychosis liability, presumably of genetic origin, direct measures rely on actual analysis of genes, their polymorphisms and their expression patterns. During the last 8 years, there has been considerable interest in the catechol-*O*-methyltransferase (COMT) gene in relation to liability to psychosis and various environmental factors including cannabis. The *COMT* gene is notable as a candidate gene for psychosis since: it is located on chromosome 22q11, a region already implicated in schizophrenia; a microdeletion of chromosome 22q11 is associated with velo-cardio-facial syndrome (which has a high rate of psychosis); and finally, the *COMT* gene codes for the enzyme catechol-*O*-methyltransferase which is involved in the metabolism of dopamine at synapses (41). From the Dunedin Study (described above), Caspi et al. (41) showed that a functional polymorphism of the *COMT* gene moderates the effect of adolescent CU on risk for adult psychosis. Homozygous carriers of the *COMT valine^158^* allele (i.e., *Val/Val*) were most likely to exhibit psychotic symptoms and later develop schizophreniform disorder if they had used cannabis during adolescence (RR: 10.9). Heterozygous individuals with the *valine/methionine (Val/Met)* genotype who had used cannabis during adolescence showed an intermediate risk; while those homozygous for the *methionine* allele *(Met/Met)* showed the lowest risk (RR: 1.1). It is important to note that the authors emphasize the fact that this effect was observed in those with adolescent-onset CU and not in those with adult-onset CU. This is important for understanding the relative impact of cannabis on the developing versus the developed brain in relation to its causal role in psychosis. Several studies have subsequently partially replicated this result (42, 43), while others have failed to replicate it (44). In a double-blind placebo controlled trial in the Netherlands, Henquet et al. (42) showed that *Val/Val* carriers were more sensitive to memory and attention impairments of delta-9-tetrahydrocannabinol (THC); although a gene-environment interaction was not demonstrated as the genotype on its own was neither associated with cognitive impairments nor associated with frequency of CU or being a patient.

Notably, *COMT* is predominantly expressed in the prefrontal cortex (45), a region associated with executive functioning, working memory, and attentional deficits in schizophrenia. The *Val/Val* genotype is associated with increased *COMT* activity and Henquet et al. (35) hypothesize that this may result in a combination of (a) reduced dopamine neurotransmission in the prefrontal cortex, and subsequently (b) increased levels of mesolimbic signaling which is thought to result in increased risk of experiencing delusions and hallucinations. Conversely, the *Met/Met* genotype is associated with better prefrontally mediated executive function performance than the *Met/Val* and *Val/Val* genotypes (46). Reasoning that since *COMT* is especially important in the prefrontal cortex, and since the prefrontal cortex is developing during puberty, Barnett et al. (47) investigated the role of *COMT* genotypes on cognitive functioning during puberty. They found that among boys who had already entered puberty, those with the *Met/Met* genotype had an average IQ 10 points higher than those with the *Val/Val* genotype. This relevant to our focus on psychosis and cannabis for two reasons: first, it supports the neurodevelopmental model of schizophrenia (48) where genetic and environmental liabilities interact with normal brain development to increase risk for the disorder (49); and secondly it provides a sound neurodevelopmental framework within which the adolescent use of cannabis can be understood as conferring increased risk for later psychosis.

## Clinical Presentation of Psychosis in Cannabis Users – are there Specific Features?

If cannabis does indeed play a causal role in psychotic illness and there is differential risk for psychosis in cannabis users, then it is pertinent to address the question of whether cannabis can be distinguished clinically as an etiology for psychosis? The view of the Indian Hemp Drug Commission over 100 years ago was that there are no distinguishing clinical features of psychosis due to CU (other than perhaps a shorter duration of the episode). Were these early impressions correct? In answering this question it is important to bear in mind the issue raised at the beginning of this review, namely: one must differentiate between lifetime use and recent/current use of cannabis as these may have quite different effects upon clinical presentation, course and outcome of psychosis.

Duration of untreated psychosis (DUP) is the period between the onset of the first psychotic symptoms and the initiation of antipsychotic treatment. DUP has significance in that longer DUP is associated with poorer response to treatment, more frequent relapses and poorer long-term outcome of psychosis (50, 51). Conversely, early detection and intervention improves outcome to a considerable degree. The popular hypothesis linking long DUP to the negative long-term consequences of psychosis relates to proposed neurotoxicity; however, an analysis of the clinical and neurobiological evidence for this hypothesis suggests that it is in fact synaptic plasticity, and not neurotoxicity, that is one of the most important mediating processes underlying this association (52). The evidence-base supporting a link between DUP and outcome is significant enough to have modified clinical practice. There is therefore a good rationale for considering whether CU impacts on DUP. A recent systematic review identified nine studies with data on DUP and CU, and meta-analysis found no significant difference in DUP between cannabis users and non-users (3). However, the author noted that six of the nine studies reported shorter DUP in cannabis users; and that “this association appears to be true specifically for samples where CU is defined in terms of current or recent use rather than lifetime use… Conversely… lifetime use appeared to be associated with longer (or in one study equivalent) DUP” (3). The author argues that this, along with other evidence to be reviewed below, suggests there may be more than one pathway to psychosis in relation to CU. In their first-episode psychosis (FEP) study, Pelayo-Terán et al. (53) compared cannabis users and non-users by *COMT* genotype and reported that those with the *Met/Met* genotype showed a relatively short DUP, irrespective of CU status (53). Those with the *Val/Val* genotype who were cannabis users also showed a short DUP, while non-cannabis users with this genotype showed statistically longer DUP. Notably, in this study, CU was defined in terms of recent use and – consistent with Burns’ conclusions cited above – cannabis users all showed shorter DUP, irrespective of their *COMT* genotype.

Early age of onset of psychosis is also associated with poorer outcome in schizophrenia. Meta-analysis shows that age of onset in cannabis users is 2.70 years earlier than in non-cannabis users; and multiple meta-regression showed that a higher proportion of cannabis users in the substance-using groups significantly contributed to the heterogeneity in the effect size (54). The authors argue that this finding lends support to the hypothesis that CU plays a causal role in the development of psychosis in some individuals. In addition, it appears that a temporal direct relationship may exist between the age at initiation of CU and age of onset of psychotic illness; with a period of 7–8 years intervening (55). In a sample of 997 individuals with psychosis, Stefanis et al. (55) found a linear association between age of initiation of CU and age of onset of psychosis; with those who had comorbid diagnoses of schizophrenia-spectrum disorder and lifetime cannabis dependence showing a slightly shorter period of premorbid cannabis exposure (7 years). A similar association has also been shown between earlier initiation of CU and early onset of high-risk symptoms for psychosis (56), suggesting that CU from a young age is associated with increased risk for a spectrum of psychotic phenomena. Thus the evidence on cannabis and age of onset of psychosis appears to support the hypothesis that early and prolonged use of cannabis is predictive of earlier onset, prognostically poorer psychotic disorder in some individuals.

Interestingly, the *COMT* genotype shows an interaction with CU in relation to age of onset (53). Pelayo-Terán and colleagues found that among non-cannabis users, age of onset was significantly later in those with the *Met/Met* genotype, compared with those homozygous for the *Val* allele. However, in cannabis users, there were no differences in age of onset between *COMT* genotypes with all cannabis users having earlier age of onset. The authors conclude that CU has the effect of reducing the delay effect of the *Met* allele on onset of psychosis; thereby depriving carriers of the relative protection conferred by this allele.

In terms of symptoms, most studies support the finding that cannabis users with FEP tend to present with more prominent positive symptoms (hallucinations, delusions, and thought disorder) and less prominent negative symptoms (apathy, social withdrawal, amotivation, etc.) (57–61). Prominent positive symptoms at onset are generally associated with a better course and outcome; while prominent negative symptoms are associated with poor outcome. Notably, Van Mastrigt et al. (57) defined CU in terms of recent use and showed increased positive symptoms in cannabis users at psychosis onset; while Compton et al. (60) reported a significant relationship between daily CU just before onset of psychotic symptoms, and an acute florid onset of psychosis. With respect to negative symptoms, Burns et al. (58) defined CU in terms of recent use and reported significantly lower negative symptom scores at psychosis onset. Taken together, these findings suggest that increased positive symptoms and reduced negative symptoms at psychosis onset are likely to be associated with the acute effects of recent/current CU specifically. This hypothesis is supported by the findings of Baeza et al. (62) who measured positive and negative symptoms at psychosis onset and again at 6 months in 32 cannabis-using and 78 non-cannabis-using children and adolescents aged 9–17 years. CU was defined in terms of recent use over the last month. At baseline (i.e., psychosis onset), cannabis users had significantly greater positive symptoms and lower negative symptoms. However, at 6 months follow-up, cannabis users had significantly lower positive and negative symptoms than non-cannabis users; particularly those cannabis users who gave up cannabis during the 6-month period. Some authors have argued that lower negative symptoms in cannabis users is a function of selection, whereby individuals with negative symptoms cannot easily access cannabis due to the apathy, amotivation, and social withdrawal that comprises the negative syndrome (59). However this is unconvincing given the ease of access to cannabis in many countries such as the Netherlands and South Africa (58).

## Cognition in Cannabis Users with Psychosis

The cognitive effects of CU in individuals with psychosis is an important and, as it turns out, fascinating topic. Long-term CU in normal individuals without psychotic disorders is associated with cognitive impairments, including residual memory and attentional deficits following abstinence (63, 64). In addition, Yücel et al. (64) have demonstrated structural brain abnormalities in otherwise healthy long-term cannabis users. However, in individuals with schizophrenia, long-term CU seems to have a different effect on neurocognitive performance. Two meta-analyses show that patients with schizophrenia who have a history of CU have superior neurocognitive functioning than patients with schizophrenia without a history of CU (65, 66). This somewhat unexpected finding appears to relate particularly to performance on executive functioning, working memory, and visual functioning; although meta-analysis of a global cognitive score also showed better performance in cannabis-using patients with schizophrenia with an overall effect size of 0.35 (66). Further analysis demonstrated that cannabis users performed significantly better (in terms of the global cognitive score) only in the studies defining CU by lifetime exposure (*d* = 0.55), but not in the studies using recent use criteria. Furthermore, Jockers-Scherübl et al. (67) found that earlier age of initiation of CU (before age 17 years) was associated with even better neurocognitive performance in patients with schizophrenia; while CU deteriorated cognitive performance in healthy controls, especially in those who initiated use before age 17 years. In a first-episode schizophrenia study, Yücel et al. (66) confirmed the finding of better neurocognitive performance in cannabis users; and replicated the finding that superior performance is specifically associated with early initiation of CU. Interestingly, more frequent CU has also been associated with better cognitive performance, specifically in the domains of working memory and attention (68).

Several explanations have been offered for this somewhat counter-intuitive finding of superior cognitive functioning in cannabis-using patients with schizophrenia. Some authors have suggested that cannabis-using patients show superior social skills, enabling them to access an illegal drug (69, 70). This however is unconvincing and is not supported by data (71). As in the case of negative symptoms (see discussion above), cannabis is readily accessible in many countries such as the Netherlands and South Africa; thus superior social skills are not necessary to obtain it.

It has also been suggested that cannabis may have a protective influence on brain functioning, especially when consumed prior to psychosis onset (67, 72). Jockers-Scherübl et al. (73, 74) found significantly higher concentrations of neurotrophins [nerve growth factor (NGF) and brain-derived neurotrophic factor (BDNF)] in schizophrenia patients with previous CU, compared to non-using schizophrenia patients. These authors suggest that “since neurotrophins like NGF and BDNF are involved in the development, plasticity, and maintenance of function of nerve cells, their up-regulation in cannabis users who later develop schizophrenia might correspond to an endogenous repair mechanism for impaired nerve cells. Cannabis might induce this mechanism, which in turn could help preserve cognitive function” (67). Supporting this hypothesis is evidence from studies of both non-clinical and other clinical (non-psychotic) populations that cannabis may have neuroprotective and even neuroregenerative properties (75–78); as well as the fact that CSF levels of the endogenous CB, anandamide, are negatively correlated with psychotic symptoms in acute untreated schizophrenia (79), suggesting an endogenous compensatory adaptation within the CB system in schizophrenia. The question of whether cannabis exerts positive neuroprotective effects on individuals who later develop schizophrenia, is likely to be controversial and highly complex and clearly further research is indicated to resolve this issue.

Perhaps, the most convincing argument for better cognitive functioning in cannabis-using schizophrenia patients is the following: those individuals who present with psychosis and a history of long-term CU, early initiation of CU increased their risk for developing psychosis, which otherwise may not have occurred in the absence of CU (66, 68, 80). In other words, early CU may induce psychosis onset in less cognitively vulnerable individuals. On the other hand, non-cannabis users who develop psychosis are likely to have greater genetic or developmental vulnerability to psychosis; thus they manifest poorer cognitive performance at psychosis onset. As Yücel et al. (66) point out, the evidence that cannabis initiation before the age of 15 years is associated with greater risk for subsequent psychosis (4, 5), supports this hypothesis. It is possible that, in the absence of cannabis, these individuals may have remained asymptomatic. Evidence that cannabis users at first-episode have fewer neurological soft signs (minor physical anomalies attributed neurodevelopmental factors) than non-users supports the hypothesis that cannabis users have less neurodevelopmental impairment (81). The fact that more frequent use of cannabis has also been associated with better cognitive performance (68), further supports this hypothesis; as here one might speculate that a greater magnitude of toxic insult is required to induce psychosis in individuals who are particularly invulnerable to psychosis.

Somewhat at odds with the hypothesis that cannabis-using schizophrenia patients have better cognitive performance and less neurodevelopmental impairment than non-users, is data suggesting structural brain abnormalities in users, especially in areas rich in CB1 receptors such as the cingulate and prefrontal cortices and cerebellum (82). In addition, two studies show reduced cortical thickness in users compared with non-users (83, 84). However, many of these studies have important limitations including: study populations are often individuals with established schizophrenia [e.g., Habets et al. (84)] and findings in these patients cannot differentiate between premorbid vulnerability markers and progressive changes during the course of the disease; and there is often comorbid alcohol or other substance use which is likely to confound results. In order to clarify this issue, first-episode samples must be studied, without other comorbid alcohol or substances. In a systematic review of the effects of CU on brain structure in schizophrenia, only four papers report on populations meeting these criteria and three of these report data from the same longitudinal cohort (82). Firstly, in Utrecht, Netherlands, Cahn et al. (85) reported from a cross-sectional FEP study no differences in global brain and caudate nucleus volumes on MRI between cannabis users and non-users. In the same group, Rais et al. (83, 86) reported no differences in ventricular size or cortical thickness at baseline in cannabis-using, first-episode schizophrenia patients; but at 5 year follow-up, those who used cannabis during the scan interval showed increased lateral and third ventricle volumes and loss of cortical thickness in the anterior cingulate cortex (ACC) and dorsolateral prefrontal cortex (DLPFC). In a different cross-sectional FEP study, Szeszko et al. (87) reported gray matter deficits in the ACC in those with a comorbid diagnosis of cannabis dependence or abuse. However, in a recent study of 54 first-episode schizophrenia patients, those with a lifetime history of CU (*n* = 30) showed higher gray matter density in the left frontal middle gyrus than those with no history of CU; and this was positively associated with better cognitive performance on the Continuous Performance Task (CPT), a measure of prefrontal cortex integrity (88). Notably, impaired performance on the CPT (in the domains of working memory, attention, and vigilance) is regarded by some as a biological trait marker for schizophrenia (89). These authors conclude that their results “provide further support for the hypothesis of a lower vulnerability for schizophrenia in at least a subgroup of cannabis-using schizophrenia patients compared to cannabis naïve patients” (88). Finally, a very recent systematic review of MRI studies in schizophrenia comparing cannabis users and non-users, reported inconclusive results, stating: “while there is some evidence that chronic cannabis abuse could alter brain morphology in schizophrenia in patients continuing their cannabis consumption, there is no convincing evidence that this alteration takes place before the onset of schizophrenia when looking at first-episode patients” (90).

In concluding this section then, it seems the strongest evidence supports the hypothesis that early CU may induce psychosis in less vulnerable individuals who otherwise may have remained well.

## Does Cannabis Use Impact on Course and Outcome of Psychosis?

While assumptions are commonly made by psychiatrists that CU impacts negatively on course and outcome of psychosis, and the seminal study of Linszen et al. (91) is often cited in support of this view, Zammit et al. (92) conclude their systematic review of the issue by stating: “We were surprised how little empirical evidence is currently available to support this view.” In their review of 13 studies that met criteria, these authors note that few studies of outcome adjust for baseline severity, and most make no adjustment for alcohol or other potentially important confounders. It is thus only worth reviewing here the findings of the three studies (93–95) that did adjust for baseline severity and for alcohol and other substance use – since these are major confounders which undermine the validity of the results of studies failing to include these adjustments. In Brisbane, a dose-response association was demonstrated between CU (days per week) and increased relapse, and in addition, increased psychotic symptoms predicted relapsed CU (93). This suggests a bidirectional relationship between CU and psychotic symptoms – thus one cannot assume that ongoing CU is causal of symptom relapse. Increased relapse of symptoms in association with cannabis was also reported from Melbourne (94) and Sydney (95), although it is important to note that neither of these studies were in FEP populations. There is also evidence that the greatest risk of relapse is associated with ongoing CU during follow-up (96). While some studies with less rigorous methodology have reported increases in positive and/or negative symptoms with ongoing CU, the only one of the three Australian studies cited above that reported increased positive symptoms is the Sydney study (95); while none found increased negative symptoms. The study of children and adolescents with FEP by Baeza et al. (62) cited earlier in this review, is notable in relation to symptoms at follow-up. Recent CU just prior to onset was associated with increased positive symptoms and lower negative symptoms. However, at 6 months follow-up, cannabis users had significantly lower positive and negative symptoms than non-cannabis users; particularly those cannabis users who gave up cannabis during the 6-month period. Thus, in conclusion, it seems Zammit et al. (92) are correct in their assessment and that there really is not any substantial evidence supporting assumptions made about poorer course and outcome in psychosis in relation to premorbid CU. Ongoing CU however appears to have a reciprocal impact on perpetuating psychosis into a possibly progressive, relapsing, deteriorating schizophrenia-like disorder.

## Pathways from Cannabis to Psychosis

Having reviewed multiple aspects of the relationship between CU and psychosis, it is now possible to propose a model which involves several pathways from cannabis to psychosis. Importantly, the factors that are key to the various pathways include;

- Early initiation/lifetime use of cannabis versus recent cannabis use.
- Underlying genetic vulnerability to psychosis/schizophrenia.
- Ongoing cannabis use after psychosis onset versus stopping cannabis use.

**Figure 1:**
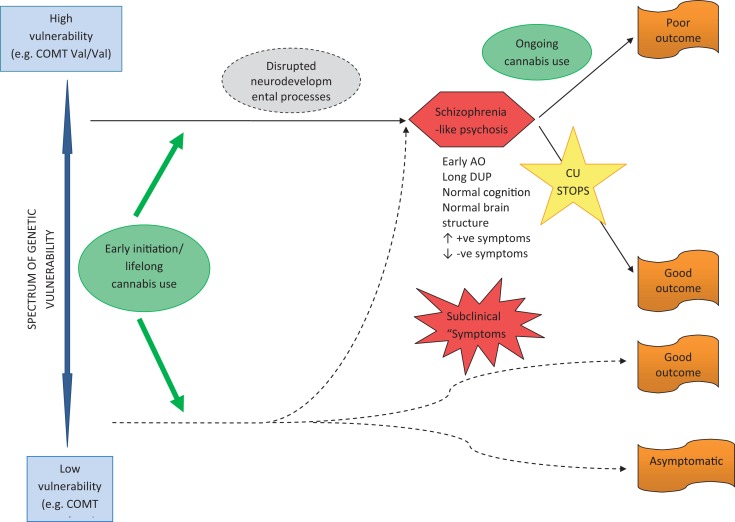
**Pathway to psychosis in early-initiated, lifelong cannabis users**.

**Figure 2:**
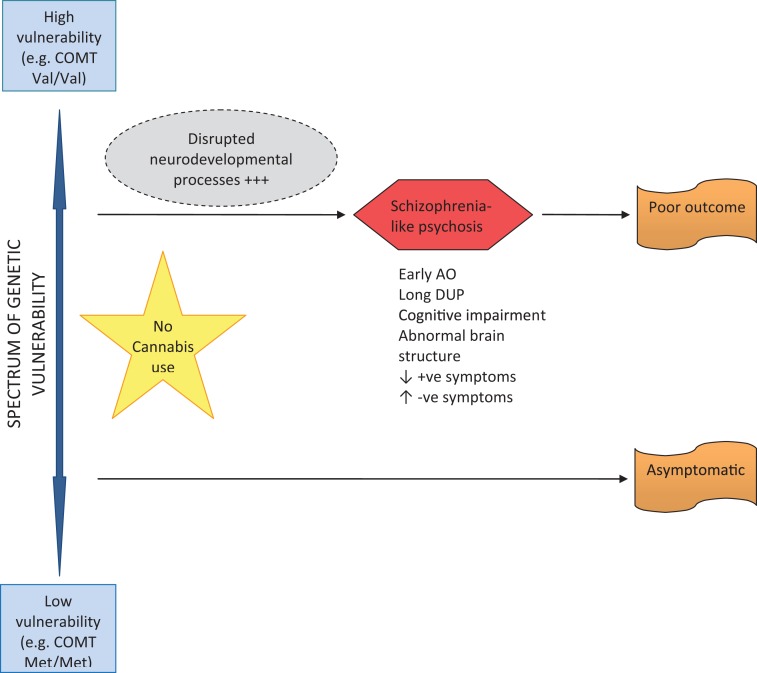
**Pathway to psychosis in non-cannabis users**.

Two major pathways from cannabis to psychosis are proposed. Firstly, early initiation of CU during adolescence and lifetime use in genetically vulnerable individuals, gives rise to neurodevelopmental changes that sensitize individuals to later psychosis – possibly through disruption of normal endocannabinoid, GABA and dopaminergic systems (see Figure 1). These are individuals who are genetically vulnerable, but in the absence of cannabis they may have remained asymptomatic. The implication is that their degree of genetic vulnerability is not as significant as that in individuals who become psychotic in the absence of CU (see Figure 2). These individuals (cannabis users) present at psychosis onset with mixed prognostic features – early age of onset, long DUP, high positive and low negative symptoms and relatively normal cognition and brain structure on MRI. If CU ceases at first-episode, then a positive outcome with significant improvement may be anticipated. However, if CU continues after psychosis onset, a poorer course and outcome characterized by repeated relapse and neurocognitive deterioration is likely. In this scenario, it appears that ongoing CU and exacerbation of psychotic symptoms impact on each other reciprocally, in a cycle of deterioration that is mirrors the underlying progression of cognitive and structural brain impairment. In such cases, the chronic deteriorating psychotic disorder is indistinguishable from schizophrenia – and in terms of current psychiatric nosology should probably be considered as schizophrenia.

The long-term outcome of early-initiated/lifelong CU in individuals who are not genetically vulnerable to psychosis, is less clear and certainly an area for future research. It is feasible that several outcomes are possible, depending on individual genetic and developmental factors, frequency, volume, and duration of CU, as well as the presence or absence of other risk factors for psychosis (such as early trauma, abuse, and stress). It is reasonable to assume that some individuals follow a course into psychosis as described above for genetically vulnerable persons; others may experience fluctuating psychotic-like symptoms that remain clinical insignificant; while others may experience a completely asymptomatic course long-term.

**Figure 3:**
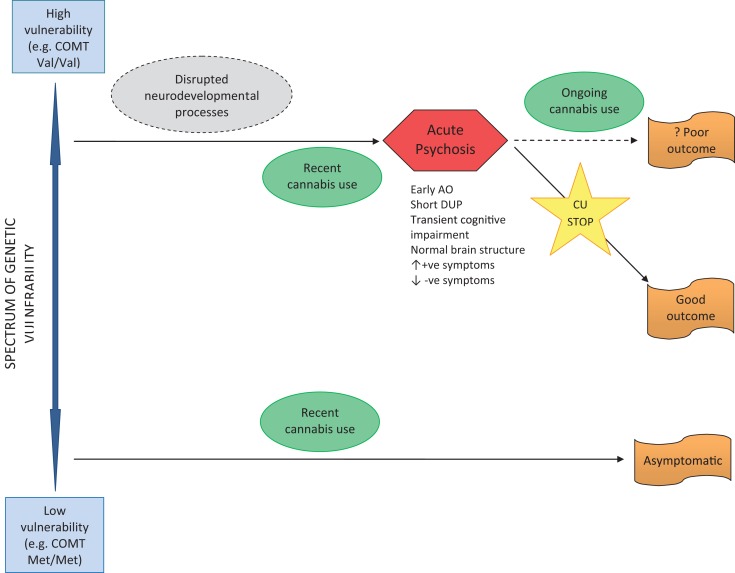
**Pathway to psychosis in recent use cannabis users**.

The other major pathway from cannabis to psychosis occurs in individuals without a lifetime history of CU, but who begin to use cannabis shortly before psychosis onset (see Figure 3). Where acute psychosis is apparently “precipitated” by recent CU, it is reasonable to assume such individuals are already genetically and developmentally vulnerable to psychosis. Thus, unlike early-initiated, lifetime cannabis users (who are vulnerable to psychosis as a result of lifelong cannabis-induced neurodevelopmental dysregulation), these individuals are susceptible to psychosis for genetic and developmental reasons alone. The acute “cannabis-induced” psychosis is characterized by prognostically better features including: later age of onset; shorter DUP; and prominent positive and absent negative symptoms. In the acute phase, cognitive deficits may be evident, but are transitory and reversible if CU ceases, with a good outcome expected. Where CU continues after psychosis onset, a poorer outcome might be expected – but this is less clear and also an area meriting further research.

A large number of people use cannabis frequently without experiencing any psychotic symptoms or disorder. Presumably, these are individuals with low genetic and developmental vulnerability to psychosis.

## Conclusion and Future Research

In conclusion, the relationship between CU and psychosis is complex and it is not possible to describe a single scenario that pertains to all individuals whose CU contributes to subsequent psychosis. In addition, variations in cannabis composition between individuals and over time are likely to contribute to heterogenous courses and outcomes. Evidence showing that the cannabis product cannabidiol (CBD) may protect against the psychosis-inducing effects of THC (97) implies that consumption of cannabis with a high THC:CBD ratio is more likely to be associated with psychotic outcomes. It is clear that at least two pathways from cannabis to psychosis exist. Early-initiated, lifelong CU in vulnerable individuals may lead to a psychotic illness virtually indistinguishable from schizophrenia at onset. It appears that outcome however is dissimilar to schizophrenia in those who cease to use cannabis after onset. In those whose CU persists, a chronic deteriorating disorder seems to follow – in these cases one may conclude that cannabis has been played a causal role in schizophrenia. Recent use of cannabis in vulnerable individuals, just prior to psychosis onset, is clinically distinguishable from schizophrenia at first-episode. Ceasing CU after the first-episode appears to have an excellent prognosis, with full recovery achievable in most cases. The long-term consequences of continued CU however are not clear.

There is clearly a need for future research to clarify and confirm these differing pathways and complex associations between CU and psychosis. Such research should be carefully designed to take into account key factors that to date have often been blurred, thereby confusing the research field. The ideal study would include the following methods:

1. A first-episode psychosis population with narrow diagnostic definitions of non-affective or schizophrenia-like psychosis.
2. A detailed history and description of CU, including: age at initiation; frequency and extent of cannabis consumed; and a clear description of patterns of recent use.
3. History of other risk factors for psychosis (e.g., childhood trauma).
4. Measures of vulnerability or proneness to schizophrenia and psychosis – these may be indirect (e.g., familial history) or direct (genetics; neuropsychological endophenotypes).
5. A longitudinal study design with clear baseline and follow-up measures of psychopathology, cognitive functioning, and ongoing CU.

Such studies will move this field forward considerably and bring us closer to answering the long-standing questions of how CU impacts on the brain and how this leads some individuals into psychotic illness and even schizophrenia. They will also clarify the issue of outcome and prognosis for individuals with a cannabis history who develop psychosis. Finally, elucidating the pathways from cannabis to psychosis will contribute substantially to ongoing efforts to understand the causes, development and natural history of schizophrenia and its related disorders.

## Conflict of Interest Statement

The author declares that the research was conducted in the absence of any commercial or financial relationships that could be construed as a potential conflict of interest.
